# The Precipitated Particle Refinement in High-Cr ODS Steels by Microalloying Element Addition

**DOI:** 10.3390/ma14247767

**Published:** 2021-12-15

**Authors:** Yingying Li, Liye Zhang, Dijun Long, Liming Yu, Huijun Li

**Affiliations:** 1 School of Materials Science & Engineering, Tianjin University, Tianjin 300354, China; li_ying_y@163.com (Y.L.); huijun@uow.edu.au (H.L.); 2 CITIC Dicastal Co., Ltd., Qinhuangdao 066000, China; imdie00@163.com; 3 Science and Technology on Reactor Fuel and Materials Laboratory, Nuclear Power Institute of China, Chengdu 610041, China

**Keywords:** ODS steel, precipitate, microstructure, tensile property

## Abstract

Two oxide-dispersion-strengthened (ODS) steels with different compositions (14Cr-ODS and 14Cr-Zr-ODS) were investigated to reveal the influences of microalloying element addition on the microstructure and to clarify the refining mechanism of precipitated particles. TEM and HRTEM results indicated that precipitated particles in the Zr-containing ODS steel had finer sizes and dispersed more homogeneously within the grains. It was found that rhombohedral Y_4_Zr_3_O_12_ particles with complex lattice structures were formed and could pin the migration of the grain boundaries during heat treatment due to their high thermal stability. In addition, the Zr-containing ODS steel exhibited a finer and more uniform grain morphology. Tensile tests showed that microalloying element addition could significantly improve the comprehensive mechanical properties of 14Cr ODS steels at room temperature.

## 1. Introduction

The rapidly growing demand for energy has pressed scientists to develop new high-temperature, high-performance structural materials for nuclear fission and fusion reactors [1,2,3]. Advanced materials can enable an improved reactor performance with increased safety margins and higher design flexibility [4,5] by providing an increased fatigue resistance, an enhanced thermal creep resistance, a superior corrosion resistance and an excellent neutron radiation damage resistance [6,7,8,9,10]. In recent years, oxide-dispersion-strengthened (ODS) steels have been considered as a promising candidate for nuclear materials, owing to their special microstructures in which nano-sized and highly stable precipitated oxide particles disperse uniformly in the matrix [11]. The existence of nano-sized oxide particles during the preparation process can contribute to the formation of finer grain size and high dislocation density, which are mainly responsible for the outstanding characteristics of ODS steels [12,13].

The manufacturing process, involving mechanical alloying (MA) of the base alloy powders with Y_2_O_3_ and subsequent sintering by hot isostatic pressing (HIP) or extrusion, is a key factor that affects the microstructure of ODS steels [14]. Additionally, the alloying design is also very crucial in determining the particle morphology, the grain size and the dislocation structure, which in turn affect the mechanical properties of ODS steels [15,16]. In addition to Fe, Cr and nano-sized Y_2_O_3_ oxides, recently developed ODS steels also introduce some other alloying elements such as W, V, Al, Ti, Zr and so on. Among them, W and V elements, as carbide formers and solid solution strengtheners, have similar effects on the microstructure and mechanical properties of the alloys; Al element can improve the corrosion resistance of ODS steels by forming a layer of dense oxide film on the surface, while it also displays a detrimental coarsening effect on the oxide particles [17]; Ti element exhibits a positive effect on refining the grain and particle sizes due to the formation of Y-Ti-O oxides [2]. Although Zr and Ti elements belong to the same subgroup in the periodic table, Zr element presents a lower activation energy for neutron capture, suggesting that the Zr-addition ODS steels possess a shorter decay time after irradiation [18]. In addition, Zr element can promote the formation of Zr-containing oxide particles that exhibit excellent thermal stability [19]. Therefore, compared with Ti element, Zr element has a more obvious optimization effect on the microstructure and mechanical properties of ODS steels. The effects of Zr addition were first introduced by Kimura et al. [20] and then confirmed by many other researchers [18,19]. In spite of this, the refining mechanism of Zr element for the microstructure of ODS steels still remains poorly understood. In addition, the evolution of microstructure affected by Zr element needs more in-depth study.

In this paper, we prepared two ODS steels of 14Cr-ODS and 14Cr-Zr-ODS to investigate the influences of Zr addition on the microstructure and mechanical properties of ODS steels. The refining mechanism of Zr addition for the microstructure, including oxide particle morphology, grain boundaries and the coherency relationship of precipitated particles with the surrounding matrix were analyzed, and the tensile properties of these two steels were also tested and discussed.

## 2. Materials and Experimental Details

Two kinds of pre-alloyed powders (<100 μm) with compositions of Fe-14Cr-1.5W and Fe-14Cr-1.5W-0.9Zr (in wt.%) were prepared using the nitrogen-gas-atomized method. Then, the two pre-alloyed powders were mixed with 0.45 wt.% Y_2_O_3_ nanoparticles (<45 nm) and mechanically alloyed by a QM-2SP12 high-energy planetary ball mill, under an argon protective atmosphere with the same parameters (a ball-to-powder weight ratio of 10:1, a rotation speed of 250 rpm and a total milling time of 30 h). Subsequently, the as-milled powders were consolidated and sintered by hot isostatic pressing (HIP) at 1150 °C under a pressure of 150 MPa for 3 h.

The microstructures were investigated by transmission electron microscopy (TEM, JEM-2100F, JEOL, Tokyo, Japan) and high-resolution transmission electron microscopy (HRTEM, JEM-2100F, JEOL, Tokyo, Japan), using a JEM-2100F operating at 200 kV. The thin foil samples used for TEM were mechanically thinned to 50 µm, then punched into discs measuring 3 mm in diameter and finally electropolished with 5% perchloric acid and 95% alcohol at −30 °C, using an MTA-1A twin jet polisher. Standard uniaxial tensile tests were carried out on a universal mechanical tester (GNT300, NCS, Shanghai, China) at room temperature with a strain rate of 1 mm/min. Tensile specimens with a gauge length of 30 mm were used in the tensile tests. For each composition, three tensile samples were tested at room temperature.

## 3. Results and Discussion

### 3.1. Effect of Microalloying Element Addition on Morphology and Distribution of Precipitated Particles in High-Cr ODS

**Figure 1:**
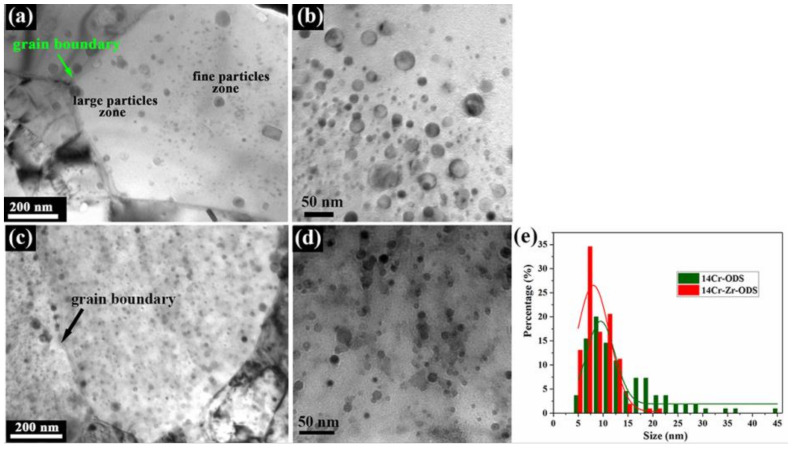
TEM bright field images of the distribution of particles in a grain, respectively, for (**a**) 14Cr-ODS and (**c**) 14Cr-Zr-ODS; TEM images of the size, density and morphology of precipitated particles at higher magnification for (**b**) 14Cr-ODS and (**d**) 14Cr-Zr-ODS; (**e**) Particle size frequency distribution histogram and Gauss fitting curves of two alloys.

Figure 1a,c show the bright field images of the distribution of nano-sized oxide particles in a grain from 14Cr-ODS steel and 14Cr-Zr-ODS steel, respectively. Figure 1b,d display the TEM images of the size, density and morphology of oxide particles at higher magnification for 14Cr-ODS steel and 14Cr-Zr-ODS steel, respectively, and Figure 1e shows the particle size frequency distribution histogram and the Gauss fitting curves of particle size in these two prepared steels. As can be seen, the shape of most oxide particles in both steels remains spherical, while the number density and distribution conditions greatly differ. As shown in Figure 1a, a typical bimodal distribution of particles sizes is detected in 14Cr-ODS steel. In such a microstructure, particles of small size (6~20 nm) are predominately precipitated within the grains, while extremely large particles are sporadically distributed around the grain boundaries and their surrounding zones. Additionally, more detailed observation reveals that the zones near the grain boundaries also contain some particle-free zones, exhibiting the absence of oxide particles. As shown in Figure 1b, the inhomogeneous distribution phenomenon of particle sizes in 14Cr-ODS steel is further confirmed, which is relatively dispersive, and some extremely large particles even have a size of 45~55 nm.

By contrast, a more dispersed distribution of particles with a smaller size can be observed in 14Cr-Zr-ODS steel, as described in Figure 1c,d. Besides, the particles distributed near the grain boundaries in 14Cr-Zr-ODS steel were finer and more homogeneous, compared with those in 14Cr-ODS steel. More than 200 particles from different TEM regions for each steel were evaluated by Image-Pro Plus (IPP, Version 6.0, Media Cybernetics, Rockville, MD, USA) software to obtain the mean diameter (*d_p_*), inter-particle distance (*λ*) and number densities of particles (*N_p_*). According to the equation: *N_p_ = N_A_/(t + d)* [21], the oxide particle volume number densities (*N_p_*) were calculated. The parameter *t* is the thickness of TEM sample and is estimated as 200 nm, suggested by [22]. *N_A_* is the area number density counted by IPP software; the values of *N_A_* are 5.75 × 10^24^ m^−2^ and 8.57 × 10^24^ m^−2^ for 14Cr-ODS and 14Cr-Zr-ODS steels, respectively. As presented in Figure 1e, the oxide particles in 14Cr-Zr-ODS have a smaller average size and a more uniform size distribution. Table 1 summarizes the statistical results of mean oxide particle diameters, average inter-particle distances and oxide particle volume number densities for two ODS steels, accounted for by TEM investigations. As Table 1 indicates, after Zr addition, the mean diameter of oxide particles decreases from 9.032 nm to 7.583 nm, the mean distance of inter-particle decreases from 19.851 nm to 15.987 nm and the number density increases from 2.75 × 10^22^ m^−3^ to 4.13 × 10^22^ m^−3^. Obviously, Zr addition can refine the mean oxide particle diameter and the average inter-particle distance of oxide particles.

### 3.2. Effect of Microalloying Element Addition on Microstructure of High-Cr ODS

In order to investigate the refining mechanism of Zr element on the microstructure of ODS steels, the formation mechanism of the inhomogeneously distributed oxide particles in 14Cr-ODS should be clarified, and the interactions between grain boundaries and oxide particles during the sintering and following cooling process should be discussed.

Many researchers have studied the evolution of yttria during the processes of mechanical alloying and sintering, by using the small angle neutron scattering (SANS) and in situ powder neutron diffraction methods [23,24]. It has been proved that yttria will dissolve in matrix powders during mechanical alloying and then the dissolved Y, O will precipitate during the subsequent hot consolidation or sintering process [25].

Based on the above dissolution mechanism, the interaction between precipitated particles and grain boundaries in the present Fe-14Cr system was investigated. During the sintering process, particle precipitation and grain boundary migration (grain growth) can occur at a high temperature combined with a high pressure. According to the Fe-Cr binary phase diagram [26], as the Cr content is higher than 12.7%, the austenitic phase transformation cannot occur. As a result, the microstructure of 14Cr-ODS in this study always exists in the form of ferrite during the heating process. According to previous studies, it has been determined that the precipitation of nano-sized oxides starts at about 600~650 °C [27], and the maximum number density of nano-sized precipitates is achieved at around 800 °C [28], while the grain growth mostly occurs over 800 °C [29]. Consequently, it can be inferred that as the grain boundaries begin to migrate, the oxide particles have been precipitated, which means that the motion of grain boundaries will interact with the precipitated particles during the sintering process at 1150 °C. Sallez et al. [30] proved that grain boundaries can dissolve nano-sized oxide particles during the migration process. This is owing to the solubility of Y in the Fe-Cr ferrite matrix above 1000 °C being as low as 0.05% [26] and the atomic radius of Y being obviously larger than that of Fe and Cr. The solubility of Y at the grain boundaries is much higher than that in the Fe-Cr matrix. As feedback, the grain boundaries will absorb Y atoms of adjacent oxide particles and result in dissolution. This statement is supported by [31,32]. In those studies, a particle-free zone formed ahead of the grain boundary. It is admitted that the solubility of alloying elements for the grain boundary is higher than that of the matrix.

**Figure 2:**
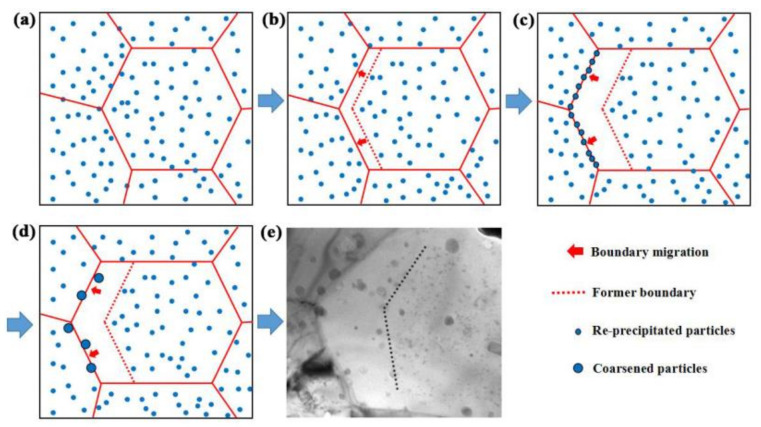
Schematic diagram of (**a**) The initial situation of grain boundaries and particles; (**b**) The process of boundary migration and particle dissolution; (**c**) The re-precipitation of the new particles at grain boundary; (**d**) The coarsening of re-precipitated particles at grain boundary; (**e**) The inhomogeneous distribution of particles caused by particle-boundary interaction in 14Cr-ODS.

Therefore, the interaction mechanism of particles and boundary can be described as follows: The migration of grain boundaries would lead to the dissolution of surrounding nanoparticles, forming a particle-depleted region behind the migrating boundaries [33]. This process can be depicted by the schematic diagrams in Figure 2a,b. As the grain boundary migration continues, the concentration of dissolved atoms around the boundaries will achieve the solubility limit and then the dissolution of oxide particles will stop and new particles begin to re-precipitate at the boundaries, as shown in Figure 2c. Meanwhile, the pinning force originating from the particles in front of the boundaries will be reinforced and then the boundary migration will soon be terminated. Finally, the particles around the boundaries will rapidly coarsen due to the enhanced diffusivity, as shown in Figure 2d. The pinning force formed by particles per unit area of grain boundary can be calculated using the following formula [34]:

(1) $$F^{\prime }=\frac{3f\gamma }{2r}$$

where *f* is the volume fraction of particles, *γ* is the interface energy per unit area and *r* is the average radius of the particles. As can be seen, it is obvious that the pinning force will be weakened with the coarsening of particles. As the pinning force is low enough compared to the driving force of boundary migration, the grain boundaries will get through the pinned particles and migrate again, leaving a region that consists of extremely large particles and blank areas behind the grain boundaries. The driving force of boundary migration comes from the energy stored in dislocations and the decrease in interface free energy. So far, the formation mechanism of large particles and blank areas near the grain boundaries of 14Cr-ODS has been clarified (Figure 2e).

**Figure 3:**
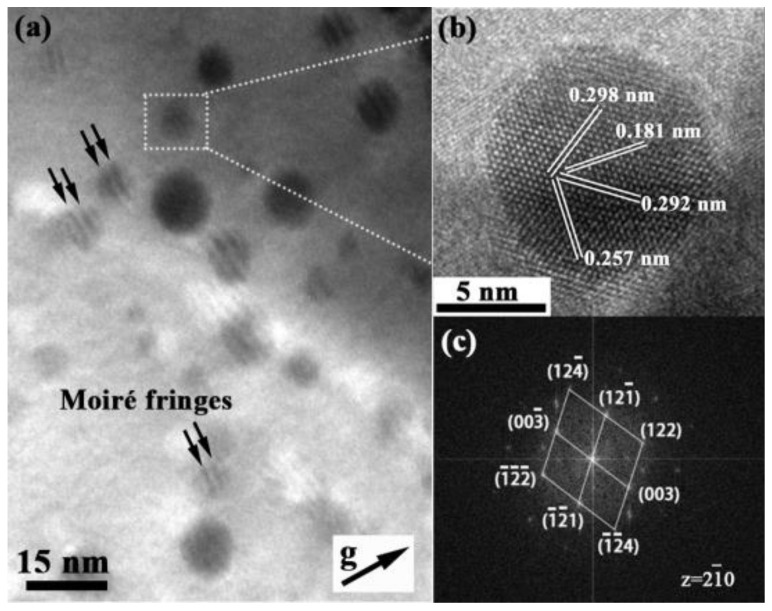
(**a**) TEM image of particles in 14Cr-Zr-ODS alloy; (**b**) HRTEM image of an Y_4_Zr_3_O_12_ particle from the area covered by the white square in (**a**); (**c**) FFT image and the index of diffraction spots of (**b**).

Figure 3a shows the TEM image of particles in 14Cr-Zr-ODS steel. It is worth mentioning that moiré fringes can be observed on most particles, indicating that most particles in 14Cr-Zr-ODS are coherent or semi-coherent with their matrix [35,36,37,38]. Our previous work [39] also discovered that the smaller the particle size, the larger spacing of the moiré fringe and the higher the coherency degree between the particles and the matrix. Hence, the existence of moiré fringes can suggest that these particles have undergone the process of nucleation and growth in some special orientations during the sintering and subsequent cooling processes. Figure 3b shows the HRTEM image of the particle selected from the area covered by the white dotted square in Figure 3a. Figure 3c shows the FFT diffraction pattern of this particle, which indicates that the inter-planar distances measured from the reciprocal space and the lattice image are very consistent with the database table for rhombohedral Y_4_Zr_3_O_12_, based on the powder diffraction pattern [40]. A number of particles in 14Cr-Zr-ODS steel with various diameters were studied by HRTEM and FFT in the present work, suggesting that the particles with relatively smaller sizes tend to exist in the Y_4_Zr_3_O_12_ phase. The formation of Y_4_Zr_3_O_12_ particles reveals the fracture of Y-O bonds and the formation of Y-Zr-O bonds. This result also demonstrated the evolution mechanism of oxide particles during the preparation process, i.e., Y_2_O_3_ particles are mechanically dissolved into the matrix during the milling and then re-precipitate during the subsequent heat treatment.

Based on the above discussion, it can be known that the nano-sized particles in the grains of 14Cr-Zr-ODS alloy are finer and are distributed much more homogeneously than those in the grains of 14Cr-ODS alloy, even in the zones near the grain boundaries. In fact, the thermal stability of Y-O particles depends on the low interfacial energy between the particles and the surrounding matrix to a large extent [37,40]. As mentioned above, Zr addition to ODS steels promoted the formation of complex rhombohedral Y_4_Zr_3_O_12_ particles. Y_4_Zr_3_O_12_ particles not only have smaller sizes but also have better coherency with the surrounding matrix, compared with Y_2_O_3_ particles. The ordered coherent interface structure between the precipitated particles and the matrix can reduce the Gibbs–Thomson effect and improve the thermal stability of the particles significantly. Therefore, it is very difficult for these particles to dissolve in the migrating grain boundaries at high temperatures. On the other hand, the first-principles calculation results show that the binding energy of Y-Zr-O clusters are higher than those of Y-O clusters in the Fe matrix, which indicates that Y_4_Zr_3_O_12_ oxides exhibit better thermal stability than Y_2_O_3_ oxides at high temperature [41]. This means that the addition of Zr improves the coarsening resistance of these particles. In summary, when particle–boundary interaction occurs during the sintering process at high temperatures, Y_4_Zr_3_O_12_ particles can exist stably and maintain their smaller sizes, eventually pinning the grain boundaries without migrating. As a result, the particles of 14Cr-Zr-ODS still disperse uniformly within the grains after the sintered alloy cools to room temperature.

**Figure 4:**
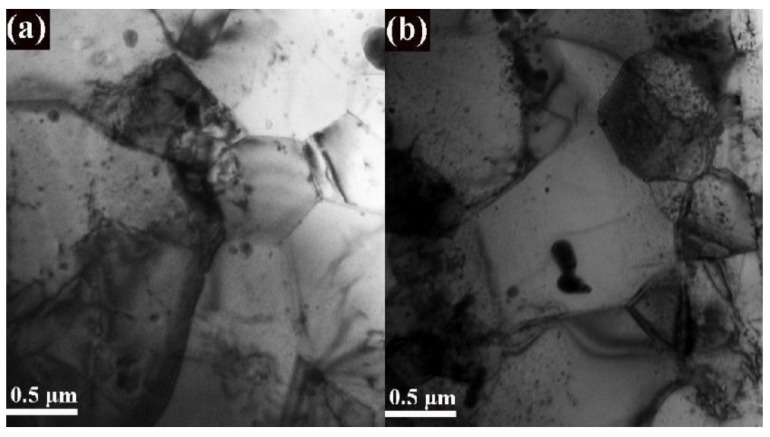
Grain morphology of (**a**) 14Cr-ODS and (**b**) 14Cr-Zr-ODS.

**Figure 5:**
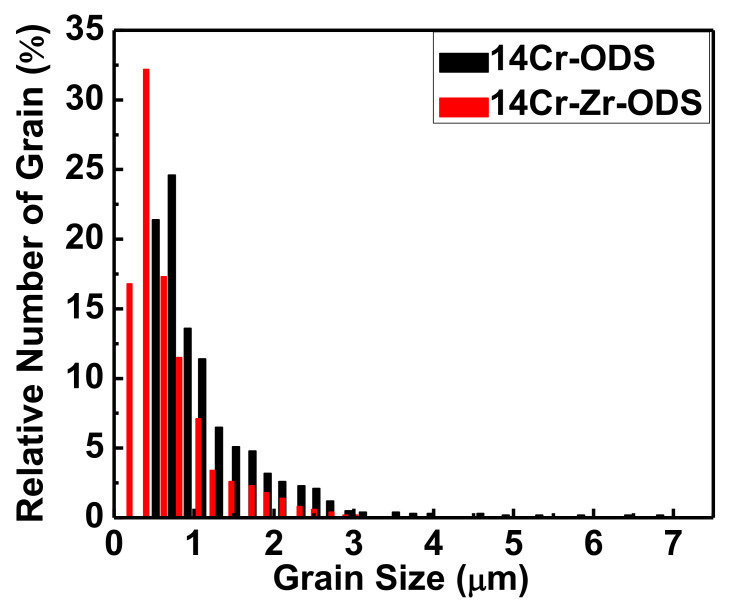
Statistical results of the grain size distribution of 14Cr-ODS and 14Cr-Zr-ODS.

Figure 4 shows the grain morphology of these two ODS steels. By using the IPP software, at least 50 TEM images were analyzed to obtain the equivalent diameter of grains. Figure 5 illustrates the statistical results of their grain size distribution. As can be seen, the grain size of 14Cr-ODS steel mainly ranges from 0.5 μm to 1.5 μm, while the grains in 14Cr-Zr-ODS (with high-density fine particles dispersed in the grain) are more homogeneous and have smaller sizes, from 0.2 μm to 0.8 μm. The higher thermal stability of the Y_4_Zr_3_O_12_ particles in 14Cr-Zr-ODS can induce a stronger pinning force on the grain boundaries and hinder their migration at high temperatures. More importantly, the superior stability of the particles in 14Cr-Zr-ODS can also prevent the abnormal growth of the grains by suppressing the reduction in the pinning force caused by particle coarsening. The statistical and calculation results show that the average grain sizes of 14Cr-ODS and 14Cr-Zr-ODS steels are 0.850 μm and 0.501 μm, respectively. Based on the above results, it can be concluded that Zr addition has an important influence on the refinement of grains in ODS steels.

### 3.3. Mechanical Properties

**Figure 6:**
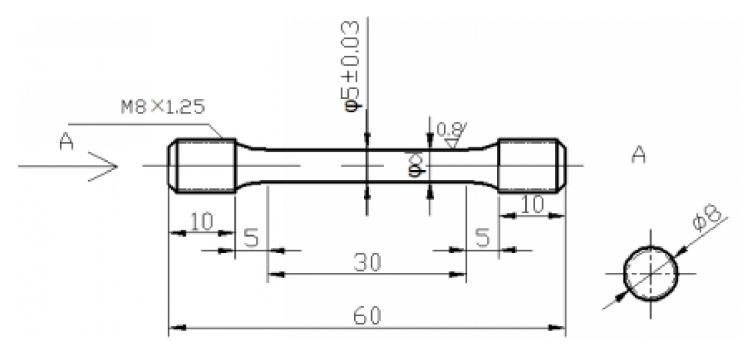
Shape and size (mm) of the tensile samples used in this work and A indicates the left view of the samples.

**Figure 7:**
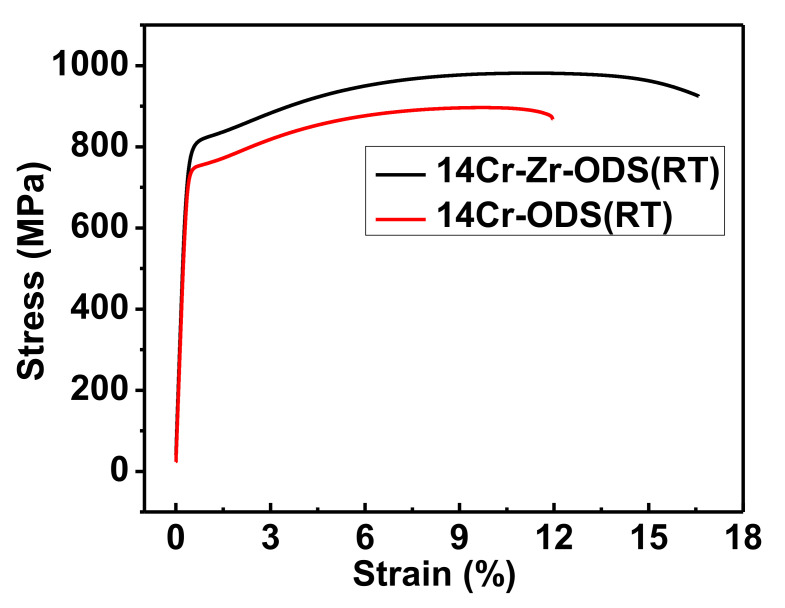
Average tensile stress–strain curves of 14Cr-ODS steel and 14Cr-Zr-ODS steel obtained at room temperature (RT).

Tensile tests for 14Cr-ODS steel and 14Cr-Zr-ODS steel were carried out at room temperature (RT), and three tensile samples were tested for each steel. The shape and size of the tensile samples are shown in Figure 6. Figure 7 shows the average stress–strain curves of three replicate tensile test results for 14Cr-ODS steel and 14Cr-Zr-ODS steel. The average yield strength (YS), ultimate tensile strength (UTS) and uniform elongation (UE) of 14Cr-ODS and 14Cr-Zr-ODS are summarized in Table 2.

As can be seen, 14Cr-Zr-ODS steel exhibits a higher yield strength and ultimate tensile strength as well as superior uniform elongation at room temperature. Thus, it can be concluded that Zr addition can not only enhance the strength but also improve the ductility of ODS steels effectively.

The superior comprehensive performance of 14Cr-Zr-ODS steel should be attributed to the optimized microstructure originating from the addition of Zr element. It is well known that there are four main strengthening mechanisms making a contribution to the yield strength in ODS steel. To explain the strengthening effects of Zr addition on ODS steels, the following equation is introduced [42,43]:

(2) $$\sigma_{y}=\sigma_{0}+\sigma_{s}+\sigma_{g}+\sigma_{d}+\sigma_{p}$$

where *σ_y_* is the yield strength of the ODS steel, *σ*_0_ is the lattice friction of pure iron, which is adopted as 53.9 MPa [42] in this paper, *σ_s_* is the contribution of solid solution strengthening, *σ_g_* is the grain size or Hall–Petch strengthening contribution, *σ_d_* is the dislocation forest strengthening contribution and *σ_p_* is the nanoparticles strengthening contribution.

The solid solution strengthening can be estimated by the equation [42]:

(3) $$\sigma_{s}=0.00689k\;X^{n}$$

where *k* is the strengthening coefficient [43] and *n* equals 0.75. *X* is the equilibrium concentration of substitutional elements in atomic percent, whose values are 15.04% and 0.46%, respectively, for Cr and W in 14-Cr-ODS, and are 15.12% and 0.46%, respectively, for Cr and W in 14-Cr-Zr-ODS. The strengthening contributions from solid solution (*σ_s_*) are calculated as 115.6 MPa and 116.2 MPa for 14-Cr-ODS and 14-Cr-Zr-ODS, respectively.

Grain refinement can result in an increase in strength and ductility simultaneously. Hence, when ODS steels are in service under high heating loads, the Y_4_Zr_3_O_12_ particles in 14Cr-Zr-ODS can possess excellent coarsening resistance and maintain their small sizes and homogeneous distribution. The refined grains in 14Cr-Zr-ODS can also make a significant contribution to the high temperature performance. As suggested in [44], the grain size or Hall –Petch strengthening contribution (*σ_g_*) can be expressed as:

(4) $$\sigma_{g}=\alpha_{g}G\sqrt{\frac{b}{d_{g}}}$$

where *α_g_* equals 0.2, *G* is the shear modulus of ferrite (80 GPa) [45], *b* is the Burgers vector of the matrix (0.248 nm) [42] and *d_g_* is the average size of grains. The estimated values of *σ_g_* are 273.3 MPa and 356 MPa for 14-Cr-ODS and 14-Cr-Zr-ODS, respectively.

The dislocation forest hardening (*σ_d_*) can be estimated by the following equation [42]:

(5) $$\sigma_{d}=\alpha_{d}MGb\sqrt{\rho_{d}}$$

Here, *α_d_* (=1/3 [44]) is an obstacle between dislocations, M is the Taylor factor which equals 3.06 for BCC metals and *ρ_d_* is the dislocation density. According to [43], the dislocation density (*ρ_d_*) is obtained from the TEM examination and the dislocation densities are 3 × 10^13^ m^−2^ and 3.4 × 10^13^ m^−2^ for 14-Cr-ODS and 14-Cr-Zr-ODS, respectively. The strengthening contributions from dislocation forest hardening (*σ_d_*) are estimated as 110.7 MPa and 118 MPa for 14-Cr-ODS and 14-Cr-Zr-ODS, respectively.

During the plastic deformation process, smaller and denser particles can produce a greater pinning effect on dislocations movements. The contribution of nanoparticles (*σ_p_*) can be estimated by the Orowan modified equation [46]:

(6) $$\sigma_{p}=\alpha_{p}MGb\sqrt{N_{p}d_{p}}$$

where *α_p_* is the obstacle strength (1/3) for nanoparticles, *N_p_* is the number density of nanoparticles and *d_p_* is the mean diameter of nanoparticles. The strengthening contributions from nanoparticles (*σ_p_*) are estimated as 290 MPa and 341.4 MPa for 14-Cr-ODS and 14-Cr-Zr-ODS, respectively.

**Figure 8:**
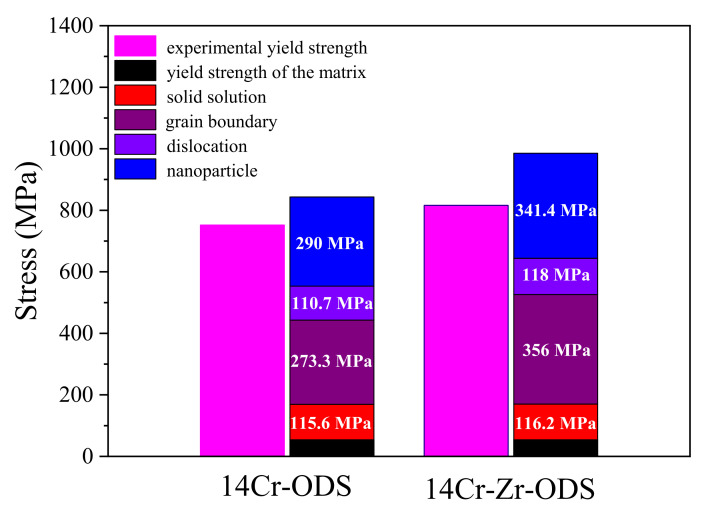
Experimental yield strengths and theoretical yield strengths represented by the components of matrix yield strength, solid solution strengthening, grain boundary strengthening, dislocation strengthening and nanoparticle strengthening of 14Cr-ODS and 14Cr-Zr-ODS steels.

Based on the values of the mean oxide particle diameters, the number density of the oxide particles and the grain sizes of these two ODS steels, the contribution of nano-sized oxide particles and grain boundaries to strengthen the yield strength can be evaluated. Based on the values of the equilibrium concentration of substitutional elements and the dislocation density of these two ODS steels, the contribution of solid solution and dislocation forest to strengthen the yield strength can be evaluated. Figure 8 shows the experimental yield strengths and the theoretical yield strengths represented by the components of the matrix yield strength, solid solution strengthening, grain boundary strengthening, dislocation forest strengthening and nanoparticle strengthening of 14Cr-ODS and 14Cr-Zr-ODS steels. It can be seen that the grain boundary and nanoparticles make a dominant contribution to strengthen the yield strength in both ODS steels. After Zr addition, σ*_g_* increases from 273.3 MPa to 356 MPa and σ*_P_* increases from 290 MPa to 341.4 MPa. Obviously, the higher grain boundary strengthening and nanoparticle strengthening of 14-Cr-Zr ODS benefit from the particle and grain refinement from Zr element addition. It is noticed that the accuracy of the results may be influenced by some statistical errors. In spite of this, the increasing trend of yield strength after Zr addition is obvious.

## 4. Conclusions

In summary, the influence of microalloying element Zr on the microstructure and mechanical properties of ODS steels were investigated, and the refining mechanism of microstructure was also discussed. Some conclusions can be drawn as follows:

(1) Zr addition into ODS steels can promote the formation of the rhombohedral Y_4_Zr_3_O_12_ phase with smaller sizes. The Y_4_Zr_3_O_12_ particles are distributed more uniformly in the matrix and have good coherency with the matrix.

(2) The Y_4_Zr_3_O_12_ particles have an excellent thermal stability due to their high diffusion resistance and coarsening resistance and can pin the migration of grain boundaries effectively.

(3) Due to the significant dispersion strengthening effect and fine grain strengthening effect, Zr addition can improve the comprehensive performance of ODS steels.

## Figures and Tables

**Table 1:** The mean oxide particle diameters (d_p_), number densities (N_p_) and average inter-particle distances (λ) of two ODS steels counted from TEM images.

	d_p_ (nm)	λ (nm)	N_p_ (10^22^ m^−3^)
14Cr-ODS	9.032 ± 1.601	19.851 ± 1.531	2.75 ± 0.21
14Cr-Zr-ODS	7.583 ± 0.825	15.987 ± 0.603	4.13 ± 0.15

**Table 2:** Comparison results of the tensile properties of 14Cr-ODS and 14Cr-Zr-ODS alloys.

	YS (MPa)	UTS (MPa)	UE (%)
14Cr-ODS	752.0 ± 22.5	892.3 ± 18.9	11.8 ± 2.1
14Cr-Zr-ODS	816.6 ± 23.1	980.6 ± 20.7	16.7 ± 1.9

## Data Availability

Not applicable.
